# Vitamin D status correction in Saudi Arabia: an experts’ consensus under the auspices of the European Society for Clinical and Economic Aspects of Osteoporosis, Osteoarthritis, and Musculoskeletal Diseases (ESCEO)

**DOI:** 10.1007/s11657-016-0295-y

**Published:** 2016-12-21

**Authors:** Nasser M. Al-Daghri, Yousef Al-Saleh, Naji Aljohani, Riad Sulimani, Abdulaziz M. Al-Othman, Hanan Alfawaz, Mona Fouda, Fahad Al-Amri, Awad Shahrani, Mohammed Alharbi, Fahad Alshahrani, Waleed Tamimi, Shaun Sabico, Rene Rizzoli, Jean-Yves Reginster

**Affiliations:** 1 Prince Mutaib Chair for Biomarkers of Osteoporosis, Biochemistry Department, College of Science, King Saud University, Riyadh, 11451 Saudi Arabia; 2 Biomarkers Research Program, Biochemistry Department, College of Science, King Saud University|, PO Box, 2455, Riyadh, 11451 Saudi Arabia; 3 College of Medicine, King Saud Bin Abdulaziz University for Health Sciences, Riyadh, 14229 Saudi Arabia; 4 Obesity, Endocrine and Metabolism Center, King Fahad Medical City, Faculty of Medicine, King Saud Bin Abdulaziz University for Health Sciences, Riyadh, 11525 Saudi Arabia; 5 Department of Medicine, College of Medicine, King Saud University, Riyadh, 11426 Saudi Arabia; 6 Sehhati National Medical Co., Riyadh, 11321 Saudi Arabia; 7 Department of Food Science and Nutrition, College of Food Science and Agriculture, King Saud University, Riyadh, 11451 Saudi Arabia; 8 Osteoporosis Control Program, Ministry of Health, Riyadh, Saudi Arabia; 9 Diabetes Centers and Units Administration, Ministry of Health, Riyadh, Saudi Arabia; 10 Department of Family Medicine, College of Medicine, King Saud Bin Abdulaziz University for Health Sciences, Riyadh, 14229 Saudi Arabia; 11 Department of Pathology and Lab Medicine, College of Medicine, King Saud Bin Abdulaziz University for Health Sciences, Riyadh, 14229 Saudi Arabia; 12 Division of Bone Diseases, Geneva University Hospitals and Faculty of Medicine, 1211 Geneva, Switzerland; 13 Department of Public Health, Epidemiology and Health Economics, University of Liège, 4000 Liège, Belgium

**Keywords:** Vitamin D, Saudi Arabia, ESCEO, Recommendations, Consensus

## Abstract

**Background:**

Vitamin D deficiency is common in the Middle East and in Saudi Arabia, in particular. While several international recommendations on the management of vitamin D deficiency have been documented and practiced globally, these recommendations should be adapted to the conditions of the Middle Eastern region. To address this challenge, the Prince Mutaib Chair for Biomarkers of Osteoporosis (PMCO) in King Saud University (KSU), Riyadh, KSA, together with local experts and in cooperation with the European Society for Clinical and Economic Aspects of Osteoporosis and Osteoarthritis (ESCEO), organized a panel that formulated unified recommendations in the diagnosis and treatment of vitamin D deficiency in the region.

**Methods:**

The selection of local and international experts commenced during the 2nd International Vitamin D Symposium conducted in Riyadh, Saudi Arabia, last January 20-–21, 2016. Reviews of the most recent literature were done, and face-to-face meetings were conducted for revisions and final recommendations.Results: Vitamin D sufficiency is defined as circulating serum 25(OH)D ≥50 nmol (≥20 ng/ml) for the general population and vitamin D adequacy as serum 25(OH)D >75 nmol/L l (>30 ng/ml) for the frail and osteoporotic elderly. Despite overwhelming prevalence of vitamin D deficiency, universal screening is not recommended. Recommendations for the general population, children, pregnant/lactating women, post-menopausal women, the elderly, and those with subsequent metabolic diseases were provided.

**Results:**

Vitamin D sufficiency is defined as circulating serum 25(OH)D ≥50 nmol (≥20 ng/ml) for the general population and vitamin D adequacy as serum 25(OH)D >75 nmol/L l (>30 ng/ml) for the frail and osteoporotic elderly. Despite overwhelming prevalence of vitamin D deficiency, universal screening is not recommended. Recommendations for the general population, children, pregnant/lactating women, post-menopausal women, the elderly, and those with subsequent metabolic diseases were provided.

**Conclusion:**

Vitamin D supplementation/correction is advised in all persons whose serum 25(OH)D falls below 50 nmol/l (20 ng/ml), and achieving a target of 75 nmol/l (30 ng/ml) is particularly suited for frail, osteoporotic, and older patients. Conducting well-designed clinical trials in the region that will address economic implications and investigations on the treatment persistence and compliance to vitamin D treatment in the region are encouraged.

## Introduction

During the last decade, no other micronutrient has gained and sustained massive interest in the fields of health and biomedical research community as much as vitamin D. As a very active research area, many of the physiologic effects of vitamin D in both mineral metabolism and extra-skeletal effects have already been covered, but not without its own share of controversies, reinforcing the need for further (despite an abundance of) investigations. Among the roster of widely debated areas in vitamin D, the cutoff used for defining vitamin D deficiency, the methods used for measuring vitamin D status and for medical practitioners as well as the informed general public, and the right treatment for vitamin D deficiency are included.

Given that the management for vitamin D deficiency remains an evolving focus in biomedical research, several well-recognized international organizations have created their own evidence-based guidelines and consensuses for the general public [1, 2], including recommendations based on harder outcomes such as the preservation of bone health [3], prevention of fractures [4], and of nutritional rickets [5]. One possible caveat from the widely used guidelines is that they were tailored mostly for the European and North American populations whose geographical location, climate conditions, culture of outdoor social activities, not to mention the ethnicity itself, vary widely from other populations. The differences in the factors mentioned, which are considered major determinants of vitamin D status, therefore cast doubt on the suitability of some international recommendations to populations coming from other regions in the world. For instance, the global consensus defines vitamin D deficiency as serum 25(OH)D < 30 nmol/l [5], while the guidelines and position statements adopted in the USA, central Europe, South America, and Korea define “deficiency” as <50 nmol/l as the more appropriate cutoff for use in their populations [6–8], twice the level adopted by the International Osteoporosis Foundation (IOF), which is <25 nmol/l [9]. The disparities in vitamin D cutoffs, not to mention the inconsistencies in terms such as deficiency, “insufficiency,” “hypovitaminosis D,” and “inadequacy,” among experts, are just a small fraction in the litany of other valid arguments (i.e., candidates for vitamin D screening and supplementation and type of 25(OH)D metabolite to measure using what method), making this seemingly straightforward, easy to treat micronutrient deficiency much more complicated. It is not surprising therefore that some national/regional societies find it most optimal to tailor the management for vitamin D deficiency in accordance to what they deem fit for their population [10, 11].

## The need for local guidelines

There is a wealth of epidemiologic evidence that vitamin D deficiency and its concomitant risk factors in the Middle Eastern region, and the Kingdom of Saudi Arabia (KSA) in particular, are very common in both children and adults [12–14]. Despite the overwhelming data, little has been done to address this epidemic on a clinical setting. Different vitamin D guidelines from other parts of the world have been produced, most of which are dependent on the target disease (osteoporosis, osteomalacia, falls, etc.) [3–6, 15–17]. The present consensus guidelines are tailored to geographical location and known risk factors common to the Saudi general population. Recommendations for the Gulf countries including Saudi Arabia, with the exception of UAE [18], are still lacking. These countries are highly reliant on other existing guidelines, which may prove to be inapplicable to regional settings. Hence, a task force was created to address the vitamin D deficiency pandemic in the country with the objective of generating a unified and tailored consensus for KSA residents and citizens.

## Prince Mutaib Chair for Biomarkers of Osteoporosis task force

The Prince Mutaib Chair for Biomarkers of Osteoporosis (PMCO) in King Saud University (KSU), Riyadh, KSA, assembled local 12 local experts in the fields of endocrinology, clinical biochemistry, and clinical nutrition and invited two advisers from the European Society for Clinical and Economic Aspects of Osteoporosis and Osteoarthritis (ESCEO), who are experts in bone diseases, health economics, and epidemiology. The selection of local and international experts commenced during the 2nd International Vitamin D Symposium conducted in Riyadh, Saudi Arabia, last January 20–21, 2016. Time frame and draft outline were discussed.

A survey was also circulated among experts to determine the experts’ consensus on the most suitable diagnostic cutoff of vitamin D deficiency to be used in Saudi Arabia, the group of populations most eligible for screening, method of measurement and type, dose and duration of supplementation depending on vitamin D deficiency severity, and other accompanying risk factors.

## Definition of vitamin D deficiency

The most common definitions used for the diagnosis of vitamin D statuses are described in Table 1. While the cutoffs vary with respect to who is vitamin D deficient, most of the major international institutions that have endorsed them are in agreement that patients whose serum 25(OH)D levels fall below 50 nmol/l (20 ng/ml) require correction [1–5, 9]. Hence, the consensus of local experts is to adapt the following cutoffs on the basis of impact on bone health:

- Deficiency—serum 25(OH)D < 50 nmol/l (<20 ng/ml).
- Sufficiency—serum 25(OH)D ≥ 50 nmol/l (≥20 ng/ml).
- Adequacy for frail, osteoporotic elderly—serum 25(OH)D > 75 nmol/l (>30 ng/ml).

**Table 1:** Most commonly used vitamin D status definitions

International agencies	Deficiency	Insufficiency	Sufficiency	Toxicity
US Endocrine Society^a^				
International Osteoporosis Foundation				
US National Osteoporosis Foundation				
American Geriatric Society				
nmol/l	<50	52.5–72.5	75	>250
ng/ml	<20	21–29	30	>100
US Institute of Medicine^a^				
UK National Osteoporosis Society				
nmol/l	<30	30–50	≥50	>125
ng/ml	<12	12–20	≥20	>50
Nordic Nutrition Recommendations^a^				
World Health Organization				
German Nutrition Society				
nmol/l	<25	<50	>50	
ng/ml	<10	<20	>20	
ESCEO^a^				
nmol/l	<25	50–75	>75	>125
ng/ml	<10	20–30	>30	>50

^a^The agency who established the definition. While definitions vary, the criteria for optimal vitamin D status have been based primarily in the following: maximal suppression of PTH, adequate intestinal calcium absorption, and fracture prevention

The recommended cutoff for vitamin D sufficiency that is much lower than the one advocated by the US Endocrine Society [25(OH)D > 75 nmol/l (>30 ng/ml)] was also based on several local observational studies that noted normal levels of PTH despite severely low levels of circulating 25(OH)D [19], lack of association between intact PTH and 25(OH)D [20], and the absence of 25(OH)D threshold at which PTH level suppression is evident [21]. This apparent confusion is also related to the target, i.e., the general population or frail, osteoporosis, possibly undernourished elderly. Nevertheless, elevated PTH levels in response to low 25(OH)D among healthy Saudis were observed only at the “insufficient” cutoff (<50 nmol/l or 20 ng/ml), although inconsistent and at varying degrees, depending on the method used [22]. This threshold for insufficiency reinforces an earlier large-scale study which was also done in the Middle-East last 2011, that a 25(OH)D threshold of 50 nmol/l is enough for PTH suppression, at least among subjects with normal renal function, for the prevention of secondary hyperparathyroidism [23]. These more recent findings supersede the earlier consensus of using 25(OH)D < 37.5 nmol/l (15 ng/ml) for the MENA region (2007) [24] and <70 nmol (28 ng/ml) (for KSA (2011) [25] as cutoffs for vitamin D insufficiency.

## Who are eligible for vitamin D screening?

Despite overwhelming evidence that vitamin D deficiency is common in the Saudi community across all age groups, vitamin D testing for the general population is not justified and practical in economic terms. Hence, the task force does not endorse population-based screening and recommends limited vitamin D testing for selected populations at highest risk for vitamin D deficiency and who will benefit the most from vitamin D status correction.

- Patients with clinical suspicion of rickets, osteopenia, osteomalacia, and osteoporosis
- Patients with history of fall and fragility fracture
- Patients with musculoskeletal pain and fibromyalgia
- Elderly patients
- Patients with malabsorption syndromes (e.g., inflammatory bowel diseases, celiac diseases)
- Patients with renal/liver diseases
- Home-based patients with minimal access to sun exposure
- Chronically ill and hospitalized patients
- Pregnant/lactating women
- Dark-skinned
- Obese
- Poor exposure to sunlight due to social/clothing habits
- Medications that affect bone bones (e.g., glucocorticoids, antiepileptic medications)
- Postbariatric surgery.

High-risk minority groups mentioned above with a confirmed diagnosis of vitamin D deficiency should receive higher than normal doses than the general population. While other non-skeletal metabolic abnormalities linked to vitamin D deficiency in the Saudi population such as obesity [26], metabolic syndrome [27], type 2 diabetes mellitus [28], and auto-immune diseases [29] may benefit from vitamin D status correction, mandatory screening is not recommended unless the patient has other risk factors and clinical features consistent with the ones mentioned above.

## Vitamin D measurement

It has been established that circulating total 25(OH)D (including both D_2_ and D_3_ forms) is the best marker for vitamin D status as compared to other metabolites. Nonetheless, with the increase in the possible number of methods to assess vitamin D status, discrepancies in the validity and reliability of results were inevitable and still exist despite efforts for standardization, making reported prevalence in a given cohort largely dependent on the method used [30–32]. Currently, the liquid chromatography–tandem mass spectrometry (LC-MS/MS) is the gold standard for measuring total 25(OH)D and other vitamin D metabolites for its high sensitivity and selectivity [33]. In major tertiary hospitals in KSA however, automated immunometric assays are preferred and major differences in results have been confirmed in local studies, mostly by Sadat and colleagues [22, 34, 35]. Well-known standardization systems include the Vitamin D External Quality Assessment Scheme (DEQAS) [36], of which PMCO in KSU is an accredited partner. The task force recommends caution in the interpretation and treatment of patient’s vitamin D status in cases where the reliability of method used is questionable, especially in the absence of other clinical features and risk factors consistent with the skeletal effects of chronic vitamin D deficiency. The task force also recommends standardization of serum 25(OH)D tests and other vitamin D metabolites in all clinical laboratories in Saudi Arabia from internationally recognized accreditation agencies such as College of American Pathologists (CAP) and DEQAS, among others.

## Vitamin D correction in the general population

### Sun exposure

Adequate sun exposure is recommended for the general population. In Saudi Arabia however, the most suitable times are as follows: summer time from 9:00 AM until before 10:30 AM as well as 2:00 PM until 3:00 PM and winter time from 10 AM to 2 PM [37], three to four times a week. People should directly expose their hands and legs to sunlight (20% of their bodies). Those with darker complexion may need more sunlight exposure time to get vitamin D. Routine monitoring of serum 25(OH)D is generally unnecessary but may be appropriate in patients with symptomatic vitamin D deficiency or malabsorption and where poor compliance with medication is suspected.

### Dietary sources and food fortification

Table 2 shows the list of vitamin D-rich foods based on the US Department of Agriculture’s Nutrient Database website [38], a strong evidence that milk and orange juice can be an effective vehicle for vitamin D fortification. Consumption of the fortified milk for 24 months significantly increased serum 25(OH)D and effectively reduced bone loss at the lumbar spine and hip [39]. Food fortification with vitamin D is submitted to the need of an efficacious quality control.

**Table 2:** Selected dietary sources of vitamin D

Food	IUs/serving	Percent of daily value
Cod liver oil, 1 tablespoon	1360	340
Swordfish, cooked, 3 oz	566	142
Salmon, cooked, 3 oz	447	112
Tuna fish, canned in water, drained, 3 oz	154	39
Orange juice fortified with vitamin D, 1 cup	137	34
Milk, nonfat, reduced fat, and whole, vitamin D-fortified, 1 cup	115–124	29–31
Yogurt fortified with 20% of the DV for vitamin D, 6 oz (more heavily fortified yogurts provide more of the DV)	80	20
Sardines, canned in oil, drained, 2 sardines	46	12
Liver, beef, cooked, 3 oz	42	11
Egg, 1 large (vitamin D is found in yolk)	41	10
Cheese, Swiss, 1 oz	6	2

*DV* daily value

### Pregnant/lactating mothers and neonates

A poor vitamin D status during pregnancy is being commonly recognized all over the world especially in the Middle East Gulf States and North African countries [24]. The World Health Organization in collaboration with the executive committee of 18th Vitamin D Workshop organized a joint symposium on the prevention and consequences of vitamin D deficiency in pregnant women and children [40]. They highlighted the importance of advice on vitamin D as a part of antenatal care. They acknowledge the lack of consensus on the required intake for this group and endorsed the Institute of Medicine (IOM) recommendation for 600 IU but encouraged research and population-based specific approaches in countries with the poorest documented vitamin D status. De-Regli and colleagues highlighted that vitamin D supplementation as routine part of antenatal care in improving maternal and fetal outcomes remain unclear, but there are inconclusive indications that correcting vitamin D status at term may reduce preeclampsia risk, low birth weight, and preterm birth [41]. In a recent systematic review that investigated the vitamin D concentrations of pregnant women in the Mediterranean region (Spain, Italy, Greece, and Turkey), up to 90% of mothers (*N* = 2649) and neonates (*N* = 1820) were deficient according to the less conservative criteria of the IOM (<50 nmol/l or <20 ng/ml) [42]. From the same cutoff, vitamin D deficiency among Saudi pregnant women ranges from 90 to 100% [43, 44].

Different recommendations and guidelines ranging from 400 IU (UK Royal College of Obstetrics and Gynecology) to 2000 IU (Canadian Academy of Pediatrics) and even 4000 IU have been documented [45]. In the case of Saudi Arabia, low vitamin D is very common in pregnancy with consequences for both mothers and babies; therefore, supplementation of 1000–2000 IU/day can be recommended and is both feasible and safe.

### Postmenopausal women

Most organizations recommend that healthy postmenopausal women over the age of 65 years should receive 800–1000 IU/day of vitamin D to maintain sufficient serum 25(OH)D levels [9]. The task force believes that this dose is suboptimal in the Gulf region. Vitamin D supplements should be used when diet and lifestyle (sunlight) recommendations cannot be implemented, which is generally the case in this class of the population.

Postmenopausal who are severely vitamin D deficient (<25 nmol/l or 10 ng/ml) can be treated with 50,000 IU of vitamin D2 or vitamin D3 once a week for 8 weeks or its equivalent of 6000 IU of vitamin D2 or vitamin D3 daily for 8 weeks followed by maintenance therapy of 1000–2000 IU/day after the serum 25(OH)D level is above 75 nmol/l. Furthermore, to sustain this level, maintenance therapy of 1500–2000 IU/D might be required [1]. Indeed, higher doses are needed in obese, postbariatric surgery, patients with malabsorption syndromes, and patients on medications affecting vitamin D metabolism at least 6000–10,000 IU/day of vitamin D [followed by maintenance therapy of 3000–6000 IU/day after the serum 25(OH)D level is above 30 ng/ml (75 nmol/l)] [1]. High doses of vitamin D would require some monitoring of 25(OH)D to avoid overdose.

### Recommendations for postmenopausal women

- Postmenopausal with severe vitamin D deficiency (<25 nmol/l or 10 ng/ml) can be treated with 50,000 IU once weekly (vitamin D2 or vitamin D3) for 8 weeks or 6000 IU/day (vitamin D2 or vitamin D3) (followed by maintenance therapy of 1000–2000 IU/day once serum 25(OH)D level is above 75 nmol/l or 30 ng/ml).
- In healthy postmenopausal women, adequate serum concentrations can be achieved through sun exposure (15 min per day, three to four times a week).
- Patients with morbid obesity, postbariatric surgery, malabsorption syndromes, and hepatic or renal diseases may require larger than usual doses of vitamin D supplements. In such patients, routine monitoring of serum 25(OH)D may be appropriate.

### Elderly and patients at risk for fall and osteoporotic fractures

Although sun exposure has been recommended to improve the status of vitamin D in the general population, this way of natural correction may not be practical in the Saudi elderly. In addition, this may be feasible only in winter months where temperatures are tolerable. Foods consumed in Saudi Arabia have little vitamin D fortification. For vitamin D deficiency, supplementation is the most guaranteed way to increase levels to the desired range of 50–75 nmol/l. This can be achieved by weekly 50,000 IU for 8 weeks followed by daily maintenance of daily 1000–2000 IU [46]. Annual mega dosing by intramuscular injections of vitamin D are not recommended as these dosage forms may not be readily available in all hospitals in KSA and their efficacy is disputed. A yearly dose of 500,000 IU has been shown to increase the risk of falls and of fractures [47].

For patients at high risk for falls and osteoporotic fractures, the same recommendations mentioned above are applicable; however, the desired 25(OH)D level should be at least 75 nmol/l (30 ng/ml). Furthermore, concomitant supplementation with calcium is advised only for those with known calcium insufficiency and those who are already undergoing treatment for osteoporosis [48].

### Patients with metabolic diseases

It has been a common feature of Saudi patients with chronic metabolic diseases (diabetes mellitus, obesity, thyroid problems, etc.) to have insufficient levels of serum 25(OH)D. Interventional studies done by Al-Daghri and colleagues in the Saudi population with metabolic disorders observed that while doses as much as 2000 IU/day for 12–18 months are still suboptimal, it did confer favorable metabolic changes in terms of improved lipid profile and insulin sensitivity [27, 49]. Jafari and colleagues in their recent meta-analysis had similar findings that vitamin D correction improves the serum lipid profile of patients with diabetes [50]. Krul-Poel et al. however has shown no conclusive evidence from gathered clinical trials that support favorable glycemic control through vitamin D supplementation among patients with diabetes [51].

In light of inconclusive observations from accumulated clinical trials, the task force therefore recommends no special consideration among patients with metabolic diseases and would manage asymptomatic vitamin D deficiency in the same case as the general population until further evidence emerges.

### Children and adolescents

Vitamin D deficiency is extremely common in the pediatric population of Saudi Arabia [52]. Adolescent females in particular have a higher prevalence of vitamin D deficiency than males and are at higher risk of osteomalacia [53].

The pragmatic use of a serum 25(OH)D concentration more than or equal to 30 ng/ml (>75 nmol/l) to indicate sufficiency and a serum concentration < 25 nmol/l (10 ng/ml) to indicate severe deficiency is recommended. All Saudi children and adolescents, females especially, are encouraged to receive oral supplementation of vitamin D as follows:

- Premature infants, 400–800 IU/day
- Infants 0–6 months, 400 IU/day
- Infants 6–12 months, 400–600 IU/day
- Children/adolescents, 600–1000 IU/day
- Obese children, 1200–2000 IU/day.

The upper limit of safety is set at

- 1000 IU/day for infants
- 2000 IU/day for children ages 1 to 10 years
- 4000 IU/day for children and adolescents ages 11–17 years.

For those in high-risk groups (preterm babies; darker color; obese; with malnutrition and malabsorption syndromes; those on medications like antiepileptic, antituberculosis, and glucocorticoids), oral supplementation of vitamin D must be considered. Universal screening for vitamin D status in the Saudi pediatric population is not advised, and monitoring should be kept at a minimum with special attention to children manifesting severe symptoms of vitamin D deficiency.

## Conclusion

The PMCO task force in cooperation with the ESCEO working group has addressed all the major aspects of vitamin D deficiency management in the Saudi population and endorses its consensus statements for general clinical practice not only in Saudi Arabia but in the Middle East in general. Vitamin D supplementation/correction is advised in all persons whose serum 25(OH)D falls below 50 nmol/l (20 ng/ml), and achieving a target of 75 nmol/l (30 ng/ml) is particularly suited for frail, osteoporotic, and older patients. The group also encourages conducting well-designed clinical trials in the region that will address risks and benefits, economic implications of vitamin D status correction and investigations on the treatment persistence, and compliance to vitamin D treatment in the region. The final recommendations assigned were based on the best available evidence at the time of submission of these guidelines to the journal.
